# Adaptive Federated Smart Homes with In-Home Displays for Mental Wellness and Sustainable Lifestyle Enhancement

**DOI:** 10.1192/j.eurpsy.2026.11346

**Published:** 2026-07-31

**Authors:** A. Oveysi Kian, M. Oveysi Kian

**Affiliations:** 1 Faculty of Electrical and Computer Engineering, Tarbiat Modares University; 2 University of Rehabilitation and Social Health Sciences, Tehran, Iran, Islamic Republic Of

## Abstract

**Introduction:**

Conventional smart home platforms focus primarily on energy efficiency and comfort optimization, often overlooking the psychosocial dimensions of well-being. Recent advances in Federated Learning (FL) and edge computing enable privacy-preserving behavioral modeling and decentralized adaptation. Within this context, In-Home Displays (IHDs) provide an interactive channel for real-time feedback, supporting self-awareness and behavioral self-regulation. However, few studies have examined how adaptive federated intelligence can actively promote mental wellness and lifestyle consistency within everyday domestic settings. This study addresses this gap by proposing an Adaptive Federated Smart Home framework that couples federated personalization with behavioral feedback loops, aiming to improve psychological indices such as the MWI, SRI, and LCS, while SEF.

**Objectives:**

The specific aims are: To design a multi-layer adaptive architecture integrating federated learning, local behavioral adaptation, and IHD-based feedback.To quantify behavioral and mental wellness improvements using normalized indices (SRI, MWI, LCS, SEF).To evaluate the feasibility, behavioral trends, and potential effect sizes in a six-week pilot study across 15 smart homes.

**Methods:**

A four-layer architecture was implemented, comprising the Cloud (Federated Aggregation), Local Adaptive Edge, Sensing, and In-Home Display Feedback layers. Each local node (smart home) learned user-specific behavioral features and fine-tuned the global federated model received from the cloud, preserving data privacy. Environmental sensors captured parameters such as light exposure, noise level, and temperature, while mood and wellness self-reports were collected through the IHD interface. The IHD also provided real-time behavioral feedback, visualizing daily metrics and trends. Behavioral indices were normalized to [0, 1], and weekly progress was evaluated across SRI, MWI, LCS, and SEF.

**Results:**

**Table 1.** Table 1. Long description.

Metric	Baseline Mean	After 6 Weeks	Change (%)
Sleep Regularity Index (SRI)	0.64	0.81	+26.6
Mental Wellness Index (MWI)	0.71	0.87	+22.5
Lifestyle Consistency Score (LCS)	0.68	0.79	+16.2
Stress Event Frequency (SEF)	0.56	0.46	−18.0

**Image 1:**
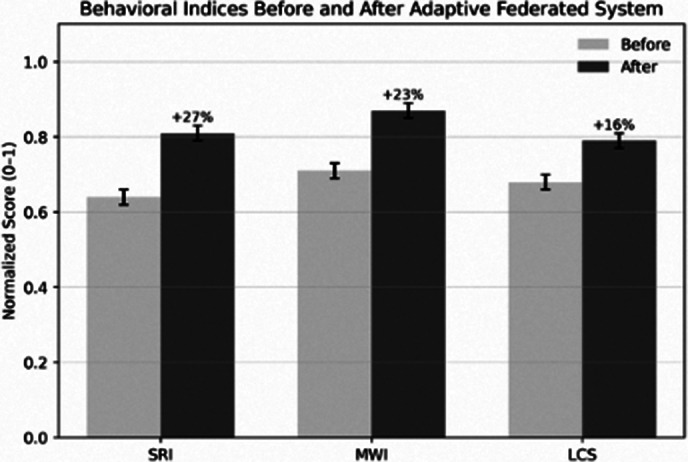
Image 1: Long description.

**Image 2:**
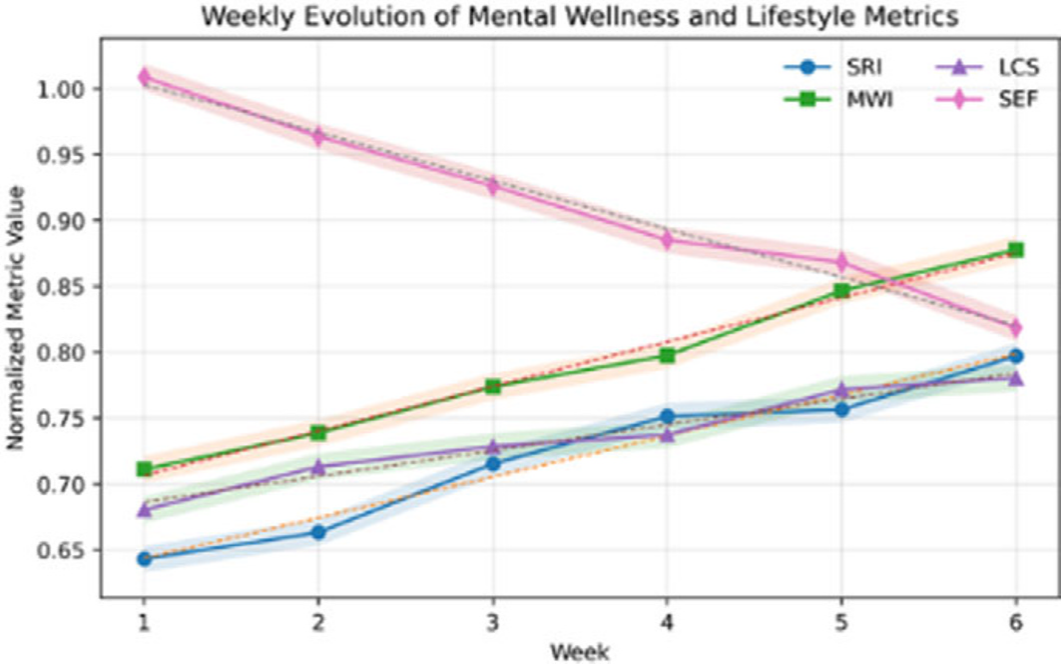
Image 2: Long description.

**Image 3:**
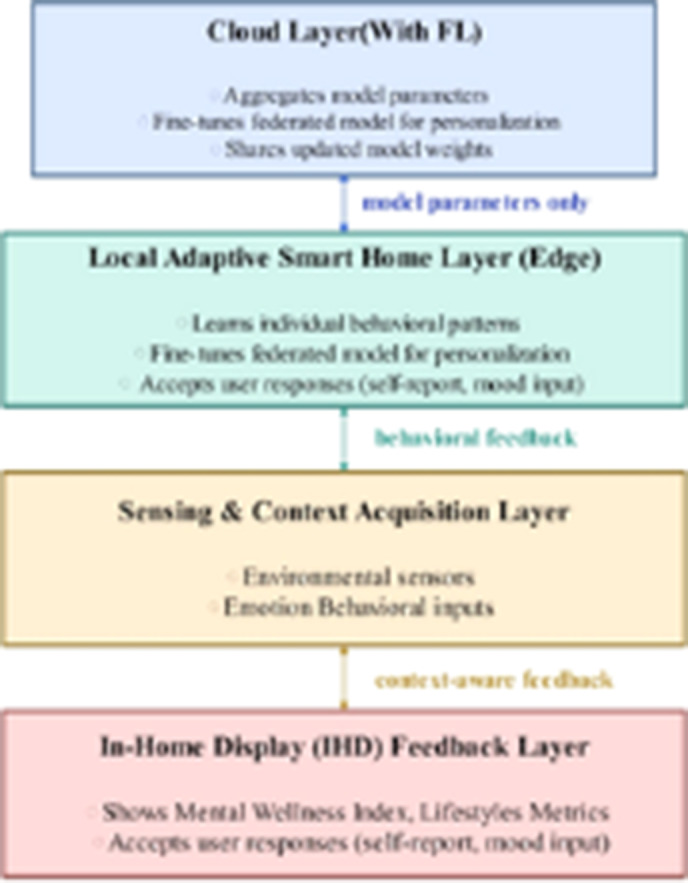
Image 3: Long description.

**Conclusions:**

This feasibility study demonstrates that adaptive federated smart homes can foster psychological well-being and sustainable living through personalized, privacy-preserving behavioral feedback. The synergy of edge intelligence and federated coordination enables local adaptation without compromising data confidentiality, offering a scalable pathway for real-world deployment.

**Disclosure of Interest:**

None Declared

## Long descriptions

### Table 1. Long description

The table consists of four columns: Metric, Baseline Mean, After 6 Weeks, and Change percentage.

* Sleep Regularity Index (S R I): Baseline Mean 0.64, After 6 Weeks 0.81, Change plus 26.6 percent.

* Mental Wellness Index (M W I): Baseline Mean 0.71, After 6 Weeks 0.87, Change plus 22.5 percent.

* Lifestyle Consistency Score (L C S): Baseline Mean 0.68, After 6 Weeks 0.79, Change plus 16.2 percent.

* Stress Event Frequency (S E F): Baseline Mean 0.56, After 6 Weeks 0.46, Change minus 18.0 percent.

Navigate back to Table 1..

### Image 1: Long description

The y-axis is labeled Normalized Score (0-1) with increments of 0.2. The x-axis lists three categories: S R I, M W I, and L C S. A legend in the top right indicates light gray bars represent Before and dark gray bars represent After. All bars include vertical error bars.

* S R I: The Before bar is at approximately 0.64. The After bar rises to approximately 0.81, labeled with a +27% increase.

* M W I: The Before bar is at approximately 0.71. The After bar rises to approximately 0.87, labeled with a +23% increase.

* L C S: The Before bar is at approximately 0.68. The After bar rises to approximately 0.79, labeled with a +16% increase.

Navigate back to Image 1:.

### Image 2: Long description

The X-axis represents Week, ranging from 1 to 6. The Y-axis represents Normalized Metric Value, ranging from 0.65 to 1.00. Four metrics are tracked with shaded confidence intervals.

* S E F (pink diamonds) shows a linear decrease, starting at approximately 1.01 in Week 1 and dropping to 0.82 by Week 6.

* M W I (green squares) shows a linear increase, starting at 0.71 in Week 1 and rising to 0.88 by Week 6. It intersects the S E F line between Week 5 and Week 6.

* S R I (blue circles) shows a steady increase from 0.64 in Week 1 to 0.80 in Week 6.

* L C S (purple triangles) shows a gradual increase from 0.68 in Week 1 to 0.78 in Week 6.

By Week 6, M W I is the highest metric, followed by S E F, S R I, and L C S. Dashed trend lines underlie each solid data line to highlight the overall trajectory.

Navigate back to Image 2:.

### Image 3: Long description

The flowchart consists of four stacked rectangular layers connected by bidirectional dashed arrows.

1. Top Layer (Blue): Cloud Layer with F L. It aggregates model parameters, fine-tunes federated model for personalization, and shares updated model weights. A blue arrow connects it to the layer below with the label: model parameters only.

2. Second Layer (Green): Local Adaptive Smart Home Layer (Edge). It learns individual behavioral patterns, fine-tunes federated model for personalization, and accepts user responses such as self-report and mood input. A green arrow connects it to the layer below with the label: behavioral feedback.

3. Third Layer (Yellow): Sensing and Context Acquisition Layer. It includes environmental sensors and emotion behavioral inputs. A yellow arrow connects it to the layer below with the label: context-aware feedback.

4. Bottom Layer (Red): In-Home Display I H D Feedback Layer. It shows Mental Wellness Index and Lifestyles Metrics, and accepts user responses such as self-report and mood input.

Navigate back to Image 3:.

